# Identifying Factors Associated with the Recurrence of Tic Disorders

**DOI:** 10.3390/brainsci12060697

**Published:** 2022-05-27

**Authors:** Yixin Zhang, Nong Xiao, Xilian Zhang, Zhenhua Zhang, Jiusi Zhang

**Affiliations:** 1 Department of Rehabilitation, Children’s Hospital of Chongqing Medical University, Chongqing 400010, China; zhangyx19951126@163.com; 2 National Clinical Research Center for Child Health and Disorders, Ministry of Education Key Laboratory of Child Development and Disorders, Chongqing 400010, China; 3 Chongqing Key Laboratory of Pediatrics, Chongqing 400010, China; 4 Chongqing Key Laboratory of Translational Medical Research in Cognitive Development and Learning and Memory Disorders, Chongqing 400010, China; 5 Department of Pediatrics, The First Affiliated Hospital of Tianjin University of Traditional Chinese Medicine, Tianjin 300380, China; zxl2072@126.com (X.Z.); zzh28920@163.com (Z.Z.); 6 Acupuncture, Tianjin Rehabilitation Center, Tianjin 300110, China; zjs19940718@163.com

**Keywords:** tic disorders, recurrence, correlation

## Abstract

Tic disorders are neurological disorders that are prone to fluctuation and recurrence. It is important to study the factors related to disease recurrence and to subsequently provide suggestions for clinical treatment. A retrospective study was conducted to assess patients with recurrent and non-recurring tic disorders diagnosed in the Pediatric Tic Disorder Clinic of the First Affiliated Hospital of Tianjin University of Traditional Chinese Medicine, China, and to extract various factors—such as fetal status; medication, allergy, and family history; social and psychological factors; blood lead content; electroencephalogram (EEG); disease duration; type of tics; and disease severity—and identify factors associated with recurrence. The recurrence rate of tic disorders was approximately 45.10% in this study. The childbirth conditions, surgery/trauma, respiratory tract infection, allergy, stress, consumption of tiapride, and severity of tic disorders were factors related to and affected disease recurrence.

## 1. Introduction

Tic disorders, manifesting as involuntary, purposeless, rapid, stereotyped muscle contractions, are neurodevelopmental disorders with increasing incidence. There are two types of tics, motor and vocal tics, which can be accompanied by a variety of comorbidities [1,2]. This disease has a long course and is prone to fluctuation and recurrence. To date, a uniform standard for the definition of the recurrence of tic disorders has not been made available in clinical practice. The reappearance of tic symptoms 3 months or longer after drug treatment completion is used as the reference for disease recurrence [3,4,5]. The recurrence of tic disorders may be related to factors such as drugs, disease duration, mental and emotional factors, and even parents’ bad habits [6,7,8]. Factors such as family disharmony, poor family education, and a lack of family affection during the process of growing up have a great impact on the aggravation and recurrence of tic disorders [9]. In addition, stress and social psychological factors also have an impact on the disease [10,11,12]. Currently, there is no consensus on recurrence-related factors, and published reports offer different conclusions. The recurrence of tic disorders brings different degrees of impact on the physical and mental health of children and enormous psychological pressure and economic burden to the parents. Hence, it is particularly important to systemically analyze and explore the factors that affect the disease recurrence and to provide recommendations for clinical treatment, thereby minimizing the recurrence of tic disorders. This study used pediatric patients who were treated in the Pediatric Tic Disorder Clinic of the First Affiliated Hospital of Tianjin University of Traditional Chinese Medicine, China. Various influencing factors, such as sex, age, fetal status, genetic factors, and disease duration, were evaluated and their impacts on disease recurrence in patients with tic disorders were assessed. The results of the present work can provide guidance for future clinical applications.

## 2. Research Objective

The primary goal of this study is to explore factors related to the recurrence of tic disorders in children treated in the Pediatric Tic Disorder Clinic to provide a preliminary strategy for reducing recurrence.

## 3. Methods

### 3.1. Criteria Section

All cases selected for this study were pediatric children treated in the Pediatric Tic Disorder Clinic of the First Affiliated Hospital of Tianjin University of Traditional Chinese Medicine in the past 5 years.

In accordance with the fifth edition of the Diagnostic and Statistical Manual of Mental Disorders published by the American Psychiatric Association [13], the patients were diagnosed with (i) transient tic disorder (TTD) if they had 1 or more motor tics and/or vocal tics, a disease duration shorter than 1 year, disease onset before 18 years of age, certain drugs and medical conditions/illnesses ruled out, and not met the diagnostic criteria of chronic tic disorder (CTD) or Tourette syndrome (TS); (ii) CTD if they had 1 or more motor tics or vocal tics, with only 1 form of tic appearing in the course of the disease, increased or decreased frequency of tics since the disease onset, a duration of the disease longer than 1 year, disease onset before 18 years of age, certain drugs and medical conditions/illnesses ruled out, and not met the diagnostic criteria of TS; (iii) TS if they had multiple motor tics and 1 or more vocal tics, both of which might not appear at the same time, increased or decreased frequency of tics, with a disease duration of more than 1 year, disease onset before 18 years of age, and certain drugs and medical conditions/illnesses ruled out.

The disease severity of the patients was defined using the Yale Global Tic Severity Scale (YGTSS) as follows: mild, with a total YGTSS score < 25; moderate, with a total YGTSS score between 25 and 50; and severe, with a total YGTSS score > 50.

The inclusion criteria for the recurrence group included meeting the diagnostic criteria of tic disorders, tic symptoms disappearing after treatment and recurring after drug withdrawal (3 months after drug withdrawal), complete clinical data, and disease onset before 18 years of age. The inclusion criteria for the control group in this study included meeting the diagnostic criteria of tic disorders, tic symptoms after the discontinuation of treatment (3 months after discontinuation), complete clinical data, and disease onset before 18 years of age.

Exclusion criteria: The exclusion criteria of this study were chorea minor, hepatolenticular degeneration, epilepsy, myoclonic seizures, drug-induced involuntary movements, and other extrapyramidal lesions; cough and other symptoms due to cold, rhinitis, pharyngitis, etc., and incomplete clinical data or loss of contact by telephone.

### 3.2. Research Section

In this study, 175 patients who received tic disorder treatment at the Pediatric Tic Disorder Clinic of the First Affiliated Hospital of Tianjin University of Traditional Chinese Medicine and had recurrence after drug withdrawal in the past 5 years were included in the recurrence group. The 175 cases were randomly selected from among 213 patients with no disease recurrence after tic disorder treatment and drug withdrawal for inclusion in the control group.

The factors included in this study were: the patients’ general information; fetal status; medical, allergy, and family history; social and psychological factors; blood lead content; electroencephalogram (EEG); disease duration; types of tics; and disease severity.

Their medical records were used as the original data. The details of the above influencing factors were also collected and were entered into an Excel spreadsheet for data sorting and analysis. The data were analyzed using SPSS statistics 26.0 statistical software. The general data and factors that were classified as “yes/no” or “normal/abnormal” were analyzed using a binary logistic regression. The chi-squared test was used to analyze count data, and the rank-sum test was used to analyze grade data. *p* < 0.05 was considered statistically significant.

## 4. Results

### 4.1. Disease Recurrence Rate

In this study, of the 388 children with tic disorders, there were 175 children with recurrence and 213 children without recurrence, showing a disease recurrence rate of approximately 45.10%.

### 4.2. Analysis of Influencing Factors for the Recurrence of Tic Disorders

For the following analyses to identify factors associated with tic disorder recurrence, we included all of the 175 children with recurrence and 175 children selected from the 213 children without recurrence.

#### 4.2.1. Correlation Analysis between Patients’ General Conditions and Disease Recurrence

As shown in Table 1, there was no significant difference between the two groups in terms of gender, age of onset, or the number of only child cases. However, in terms of the physical condition of the children at birth, there were 14 cases (8.0%) in the recurrence group and 5 cases (2.9%) in the control group were in poor physical condition.

Binary logistic regression analysis of the general conditions of the two groups of patients showed that the health status at birth of the patients was significantly different between the two groups (*p* < 0.05).

#### 4.2.2. Correlation Analysis between Birth Status and Disease Recurrence

By comparing the abnormal fetal birth of the mothers of the two groups, we found that the highest proportion in the recurrence group was 11 cases (20%) of elderly parturient, 10 cases (18.2%) of abnormal fetal position, and 7 cases (12.7%) of medication during pregnancy. The highest proportion in the control group was nine cases (28.1%) of elderly parturient, five cases (15.6%) of emotional disorders, and five cases (15.6%) of pregnancy-induced hypertension.

Binary logistic regression analysis was performed on the birth status of the mothers of the pediatric patients and showed that abnormal fetal position was associated with recurrence (*p* < 0.05; Table 2).

#### 4.2.3. Correlation Analysis between Medical and Allergy History and Disease Recurrence

As shown in Table 3, in an analysis of past medical history and allergies in the two groups, 24 cases (13.7%) in the recurrence group and 9 cases (5.1%) in the control group had respiratory tract infections before the onset, and 30 cases in the recurrence group had respiratory tract infections before the recurrence. A total of 32 cases (18.3%) in the recurrence group and 13 cases (7.4%) in the control group had a history of food and drug allergies. A total of 30 cases (29.1%) in the recurrence group (103 cases had surgical/ trauma and other diseases except respiratory infection) and 8 cases (16.7%) in the control group (48 cases had surgical/ trauma and other diseases except respiratory infection) had conjunctivitis. There were 12 cases (11.7%) of operation and trauma in the recurrence group and 2 cases (4.2%) in the control group.

The binary logistic regression analysis of the medical and allergy history of the patients showed that respiratory tract infection, conjunctivitis, surgery/trauma, and history of food and drug allergy were associated with recurrence (*p* < 0.05).

#### 4.2.4. Correlation Analysis between Family Medical History, Blood Lead Content, EEG, and Disease Recurrence

In the analysis of the family histories of the two groups, it was found that, among the close relatives of the recurrence group (25 cases had family medical history) and the control group (18 cases had family medical history), the number of cases with tic disorders was the highest, with 15 cases (60.0%) and 12 cases (66.7%), respectively.

As shown in Table 4, the binary logistic regression analysis of family history of tic disorders, epilepsy, mental disorders, blood lead content, and EEG abnormalities showed no significant correlations with disease recurrence (*p* > 0.05).

#### 4.2.5. Correlation Analysis between Psychosocial Environmental Factors and Disease Recurrence

In the psychosocial analysis of the two groups, the recurrence group and the control group all had the most cases of incorrect parental education. In addition, the number of cases with emotional stimulation, stress, and fatigue in the recurrence group was significantly higher than that in the control group.

As shown in Table 5, the specific factors of the psychosocial environment were analyzed by binary logistic regression and showed that emotional stimulation, stress/fatigue, and excessive viewing of electronic devices (too much screen time) were associated with recurrence (*p* < 0.05).

#### 4.2.6. Correlation Analysis between the Type of Medication and Disease Recurrence

In a comparison of medication use between the two groups, the combined medication use frequency in the recurrence group was higher than that in the control group. In the recurrence group, 58 cases took tiapride and 21 cases took aripiprazole. In the control group, 21 cases took tiapride and 10 cases took aripiprazole.

A binary logistic regression analysis of different medications was performed to determine whether different types of medication affected the recurrence of tic disorders. The results show that the consumption of tiapride was significantly correlated with disease recurrence (*p* < 0.05, Table 6).

#### 4.2.7. Correlation Analysis between Disease Duration, Tic Classification, Disease Severity, and Disease Recurrence

In terms of disease duration, both groups had the largest number of children with a disease duration of less than one year, with 59 cases (33.7%) in the recurrence group and 67 cases (38.3%) in the control group. The number of TS in the recurrence group (85 cases, 48.6%) was higher than that in the control group (73 cases, 41.7%). In terms of disease severity, there were 54 mild cases (30.9%) in the recurrence group compared to 73 cases (41.7%) in the control group; while there were 114 moderate cases (65.1%) in the recurrence group compared to 96 cases (54.9%) in the control group.

To explore the relationship between disease duration, tic classification, disease severity, and disease recurrence, the rank-sum test (disease duration and disease severity) and chi-squared test (tic classification) were performed and showed a significant correlation between disease severity and recurrence (*p* < 0.05, Table 7).

## 5. Discussion

### 5.1. Disease Recurrence Rate

A review of the relevant literature found that most of the literature on the recurrence rate of tic disorders was based on short-term follow-ups in self-controlled trials. Due to the influence of drugs, treatment methods, and treatment cycles, the disease recurrence rates of the different studies varied greatly, generally ranging from 20% to 60% [5,14]. The disease recurrence rate in this study was 45.10%, a relatively high level.

### 5.2. The Results of Correlation Analysis of Various Factors and Disease Recurrence

Patients’ general conditions and disease recurrence: Health at birth can affect the recurrence of tic disorders. The specific diseases of the two groups of patients with poor health at birth were analyzed, showing that there were 10 diseases at birth in the recurrence group and 3 diseases in the control group. Among all the children with poor health at birth, the proportion of neonatal jaundice was high (three cases in each group). However, it was not found to be a risk factor for disease recurrence, possibly due to the relatively small sample size in this study. A future univariate cohort analysis with a larger sample size could be conducted to test whether neonatal jaundice is a risk factor for disease recurrence.

Birth status and disease recurrence: Abnormal fetal position during pregnancy can affect the recurrence of tic disorders. A previous study also indicated that an abnormal fetal position may affect the health status of children at birth, thereby affecting disease recurrence [15].

Medical and allergy histories and disease recurrence: A history of respiratory tract infection, conjunctivitis, surgery/trauma, and food and drug allergies can affect the recurrence of tic disorders. A previous study showed that abdominal twitching in children may be related to the gastrointestinal symptoms caused by respiratory tract infections, and this may further affect the prognosis of the disease [16]. Some studies indicated that hyperactivity and allergic factors are associated with disease recurrence [17]. In this study, allergy affected the disease recurrence, while no correlation was found between hyperactivity and disease recurrence, which may be because the pediatric patients had comorbid hyperactivity during the treatment of tic disorders, and this active treatment controlled the hyperactivity well. A clinical univariate cohort analysis may be performed in the future to analyze patients with tic disorders with and without comorbid hyperactivity and study the correlation between disease recurrence and hyperactivity.

The family medical history and disease recurrence: The children’s close relatives that suffered from tic disorders, mental disorders, and epilepsy were not associated with the recurrence of tic disorders. Studies have shown that a family history of tic disorders can be used as a risk factor for recurrence [17,18,19]. However, this study did not support this conclusion, and this may have been due to the fact that the parents of children with a family history of tic disorders took relevant precautions, which may have affected the prognosis of the disease.

Psychosocial environmental factors and disease recurrence: Emotional stimulation, stress/fatigue, and too much screen time can affect the recurrence of tic disorders. Studies have shown that some psychosocial environmental factors, such as emotional stimulation, learning stress/fatigue, and family education, are related to the recurrence of tic disorders [3,20,21], and this is consistent with the findings of this study. However, no correlation was found between the family factors and the disease recurrence in this study. This may be because the families received information on how family education affects the disease after the children were diagnosed with tic disorders, and some parents subsequently changed their parenting style, which may have reduced the stress and worry of the children, thereby improving the disease.

The type of medications and disease recurrence: Tiapride may be one of the risk factors for the recurrence of tic disorders. This significant correlation may be due to some children in the recurrence group of this study being more seriously ill, and the proportion of tiapride consumption of the recurrence group being significantly higher, compared with the control group.

Disease duration, tic classification, disease severity, and disease recurrence: The duration of the disease was not associated with the type of tic, and the severity of the tic disorder was related to the recurrence. Studies have shown that patients with mild tic disorders have a better prognosis than patients with moderate-to-severe tic disorders, and they are less likely to have disease recurrence [5,22,23], which is consistent with our findings. However, our findings did not agree with previous research, indicating that the longer the duration of tic disorders, the higher the recurrence rate [20]. Our analysis showed that some children in the recurrence group had frequent tic symptoms at disease onset and received more active treatment. The disease was well controlled in a relatively short period of time in the recurrent group, while some patients in the control group had less frequent tic symptoms, and their compliance and cooperation with the treatment were worse than those in the recurrence group, leading to a relatively prolonged illness. Therefore, the difference between the two groups of patients was not obvious.

## 6. Conclusions

In this study:

(1) The recurrence rate of tic disorders was approximately 45.10%.
(2) Threatened miscarriage during pregnancy, advanced maternal age, umbilical cord around the neck, majority pregnancy and perinatal issues, age of onset, singleton status, rhinitis, febrile convulsion, hyperactivity, epilepsy, encephalitis, blood lead, and EEG; whether close relatives suffer from tic disorders, mental disorders, and epilepsy; improper upbringing, parental quarrel/divorce, environmental change, disease duration, and disease classification were not associated with the recurrence of tic disorders.
(3) Abnormal fetal position during pregnancy, poor health at birth, respiratory infection, conjunctivitis, surgery/trauma, food and drug allergy, emotional stimulation, stress/fatigue, too much screen time, tiapride consumption, and disease severity were risk factors for the recurrence of tic disorders.

## 7. Difference

Currently, the research on tic disorders is primarily based on its pathogenesis, drugs, and psychobehavioral therapy and pays less attention to the factors of recurrence. The recurrence rate of tic disorders is high, and the factors causing recurrence are complex and diverse. In this study, various possible factors that may cause recurrence were explored. This study provides references for clinical application.

## 8. Suggestions

Physicians should pay attention to the prevention of exogenous diseases and of the occurrence and recurrence of conjunctivitis in children with tics. In addition, children with food and drug allergies should avoid allergens in their daily life. The parents of pediatric patients should encourage a balance between study and rest/relaxation, without putting too much pressure and stimulation on the children, and should ensure that the children do not spend too much time on electronic devices. Any tics or tic-like symptoms in pediatric patients should not be ignored, regardless of their severity, and should be treated as soon as possible.

## Figures and Tables

**Table 1:** Correlation between general information and tic recurrence (non-modifiable factor).

General Information	Various Categories	Recurrence Group, *n* (%)	Control Group, *n* (%)	*OR*	*p*
Sex	Male	139 (79.4%)	138 (78.9%)	1.001	0.996
	Female	36 (20.6%)	37 (21.1%)		
Age of onset	0–6 years old	119 (68.0%)	118 (67.4%)	0.953	0.841
	7–12 years old	54 (30.9%)	54 (30.9%)		
	13–18 years old	2 (1.1%)	3 (1.7%)		
Only child	Yes	143 (81.7%)	146 (83.4%)	1.120	0.691
	No	32 (18.3%)	29 (16.6%)		
Health at birth	Great	161 (92%)	170 (97.1%)	2.932	0.044 *
	Poor	14 (8.0%)	5 (2.9%)		

* indicates: *p* < 0.05, The difference had statistically significant.

**Table 2:** Relationship between birth status and tic recurrence (non-modifiable factor).

Fetal Status at Birth	Related Factors	Recurrence Group, *n* (%)	Control Group, *n* (%)	*OR*	*p*	
Abnormalities during pregnancy	Elderly parturient	11 (20.0%)	9 (28.1%)	1.165	0.747	
	Abnormal fetal position	10 (18.2%)	2 (6.3%)	5.057	0.043 *	
	Medication during pregnancy	7 (12.7%)	3 (9.4%)	1.905	0.385	
	Threatened miscarriage	7 (12.7%)	3 (9.4%)	2.338	0.233	
	Mood disorders	6 (10.9%)	5 (15.6%)	0.846	0.800	
	Abnormal amniotic fluid	5 (9.1%)	3 (9.4%)	1.363	0.689	
	Gestational diabetes	3 (5.5%)	1 (3.1%)	2.442	0.455	
	Malnutrition during pregnancy	3 (5.5%)	1 (3.1%)	3.246	0.321	
	Gestational hypertension	3 (5.5%)	5 (15.6%)	0.614	0.514	
Perinatal abnormalities	Nuchal cord	39 (70.9%)	30 (83.3%)	1.471	0.156	
	Premature rupture of membranes	12 (21.8%)	5 (13.9%)	2.539	0.090	
	Vacuum extraction, forceps delivery	4 (7.3%)	1 (2.8%)	3.958	0.225	
Term of pregnancy	Full-term birth	162 (92.6%)	166 (94.9%)	1.252		0.627
	Premature birth	11 (6.3%)	9 (5.1%)			
	Post-term birth	2 (1.1%)	0 (0%)			
Delivery method	Vaginal delivery	62 (35.4%)	58 (33.1%)	0.958		0.851
	Cesarian section	113 (64.6%)	117 (66.9%)			

* indicates: *p* < 0.05, The difference had statistically significant.

**Table 3:** Correlation of other diseases and allergic factors with tic recurrence (modifiable factor).

Related Factor	Recurrence Group, *n* (%)	Control Group, *n* (%)	*OR*	*p*
Respiratory infection (before onset)	24 (13.7%)	9 (5.1%)	2.926	0.012 *
Respiratory infection (before relapse)	30 (17.1%)	9 (5.1%)	3.816	0.001 *
Allergy history (food and drug)	32 (18.3%)	13 (7.4%)	2.882	0.003 *
Rhinitis	18 (17.5%)	9 (18.8%)	1.844	0.175
Conjunctivitis	30 (29.1%)	8 (16.7%)	4.086	0.001 *
Febrile seizures	7 (6.8%)	7 (14.6%)	0.968	0.956
Hyperactivity	28 (27.2%)	18 (37.5%)	1.666	0.133
Epilepsy	5 (4.9%)	2 (4.2%)	3.408	0.156
Encephalitis	3 (2.9%)	2 (4.2%)	1.962	0.473
Surgery/trauma	12 (11.7%)	2 (4.2%)	6.033	0.024 *

* indicates: *p* < 0.05, The difference had statistically significant.

**Table 4:** Correlations between family medical history, blood lead, EEG, and tic recurrence (modifiable factor).

Related Factor	Recurrence Group, *n* (%)	Control Group, *n* (%)	*OR*	*p*
Tic disorders in relatives	15 (60.0%)	12 (66.7%)	1.253	0.577
Mental disorders in relatives	6 (24.0%)	2 (11.1%)	3.072	0.175
Epilepsy in relatives	4 (16.0%)	4 (22.2%)	0.912	0.899
Abnormal blood lead	7 (4.0%)	2 (1.1%)	3.235	0.150
Abnormal EEG	11 (6.3%)	5 (2.9%)	2.079	0.189

**Table 5:** Relationship between psychosocial and environmental factors and tic recurrence (modifiable factor).

Related Factor	Recurrence Group, *n* (%)	Control Group, *n* (%)	*OR*	*p*
Improper parenting/excessive discipline	58 (33.3%)	63 (47.7%)	0.839	0.462
Divorced and fighting parents	10 (5.7%)	10 (7.6%)	1.086	0.862
Parents not around	3 (1.7%)	3 (2.3%)	0.988	0.989
Emotional stimulation	22 (12.6%)	10 (7.6%)	2.700	0.015 *
Stress and fatigue	17 (9.8%)	6 (4.5%)	3.421	0.013 *
Too much screen time	14 (8.0%)	4 (3.0%)	4.663	0.008 *
After trauma and illness	7 (4.0%)	2 (1.5%)	4.408	0.069
Renovation	12 (6.9%)	9 (6.8%)	1.010	0.985
Moving	20 (11.5%)	18 (13.6%)	1.225	0.597
Environmental changes	11 (6.3%)	7 (5.3%)	1.665	0.327

* indicates: *p* < 0.05, The difference had statistically significant.

**Table 6:** Correlation between several types of medication and tic recurrence (modifiable factor).

Medication	Recurrence Group, *n* (%)	Control Group, *n* (%)	*OR*	*p*
Tiapride	58 (56.9%)	21 (53.8%)	2.249	0.002 *
Aripiprazole	21 (20.6%)	10 (25.6%)	2.140	0.064
Sertraline	7 (6.9%)	1 (2.9%)	6.035	0.099
Haloperidol	16 (15.7%)	7 (17.9%)	2.404	0.065

* indicates: *p* < 0.05, The difference had statistically significant.

**Table 7:** Correlation between disease duration, tic classification, disease severity, and recurrence of tic disorders (modifiable factor).

Factor		Recurrence Group, *n* (%)	Control Group, *n* (%)	*Z*/*χ*^2^	*p*
Disease duration	<1 year	59 (33.7%)	67 (38.3%)	−0.133 (*Z*)	0.894
	1 to 2 years	58 (33.1%)	40 (22.9%)		
	2 to 3 years	23 (13.1%)	35 (20.0%)		
	>3 years	35 (20.0%)	33 (18.9%)		
Classification of tic disorders	TTD	59 (33.7%)	67 (38.3%)	1.662 (*χ*^2^)	0.436
	CTD	31 (17.7%)	35 (20.0%)		
	TS	85 (48.6%)	73 (41.7%)		
Disease severity	Mild	54 (30.9%)	73 (41.7%)	−2.040 (*Z*)	0.041 *
	Moderate	114 (65.1%)	96 (54.9%)		
	Severe	7 (4.0%)	6 (3.4%)		

* indicates: *p* < 0.05, The difference had statistically significant.

## Data Availability

Data are contained within the article.
